# Efficacy and safety of sorafenib combined with transarterial chemoembolization in the treatment of hepatocellular carcinoma: a meta-analysis of randomized controlled trials

**DOI:** 10.3389/fonc.2025.1640879

**Published:** 2025-11-10

**Authors:** Lixiu Yu, Yi Xiong, Jing Liao, Yahui Deng, Xuejia Zhai

**Affiliations:** 1 Department of Pharmacy, Union Hospital, Tongji Medical College, Huazhong University of Science and Technology, Wuhan, China; 2 Hubei Province Clinical Research Center for Precision Medicine for Critical Illness, Wuhan, China; 3 University of Wuhan, Wuhan, China

**Keywords:** hepatocellular carcinoma, sorafenib, transarterial chemoembolization, randomized controlled trials, meta-analysis

## Abstract

**Objective:**

Transarterial chemoembolization (TACE) plus sorafenib has led to an increase in randomized controlled trials The efficacy and safety of sorafenib combined with TACE for the treatment of hepatocellular carcinoma (HCC) remain controversial. We conducted a comprehensive meta-analysis of randomized controlled trials on this issue.

**Methods:**

A literature search was conducted by using online database: PubMed, the Cochrane Library, Embase, Web of Science, Chinese National Knowledge Infrastructure (CNKI) and Wan-fang, with no language restrictions. A Meta-analysis was performed to calculate the pooled odds ratio (OR) and its corresponding 95% confidence interval (CI) for the efficacy and safety of sorafenib combined with TACE in the treatment of HCC. Review Manager 5.4 software was used for data analysis.

**Results:**

A total of 19 randomized controlled trials involving 2,029 patients met the inclusion criteria for this meta-analysis, including sorafenib combined with TACE group (n=1023) and TACE group (n=1006). The results of meta-analysis showed that sorafenib combined with TACE had a better prognosis in partial response rate (PR) [OR = 1.58,95%CI (1.30,1.92), P < 0.00001] with low heterogeneity among studies (P = 0.67; I2 = 0%) and the objective response rate (ORR) [OR = 1.93, 95%CI (1.59,2.34), P < 0.00001] with low heterogeneity among studies (P = 0.42; I2 = 3%). The 12-month overall survival (OS) was also significantly increased by combination therapy [OR = 3.18, 95%CI (2.41,4.19), P < 0.00001]. In terms of safety, the incidences were significantly high in TACE plus sorafenib group compared to TACE group for hand-foot skin reaction OR = 4.48, 95%CI (3.28,6.13), P < 0.00001 and for abdominal pain or diarrhea OR = 3.10, 95%CI (2.24,4.29), P = 0.04. However, no significant difference was found in nausea or vomit [OR = 1.14,95%CI (0.81,1.59), P = 0.68] or fever [OR = 0.87, 95%CI (0.61,1.23), P = 0.87].

**Conclusion:**

The current comprehensive evidence suggests that sorafenib combined with TACE is more effective than TACE alone, especially for patients with intermediate-advanced and primary HCC.

## 1 Introduction

Hepatocellular carcinoma (HCC) is the most common types of primary malignant tumor of the liver, posing a significant threat to human health (1). The treatment of HCC presents challenges due to the disease’s complexity, which is typically diagnosed at an advanced stage and often develops in the context of cirrhosis (2). The treatment methods for liver cancer include surgical resection, liver transplantation, local ablation, transarterial embolization, and transarterial chemoembolization (TACE). Surgical resection and liver transplantation are considered as radical treatment methods for liver cancer. However, due to patients often with severe liver function impairment, the surgical resection rate remains relatively low. Local ablation techniques, such as radiofrequency ablation and microwave ablation, are appropriate for patients with small hepatocellular carcinomas but show limited effectiveness for larger tumors. Consequently, for patients with intermediate to advanced liver cancer, TACE is often employed as a local interventional therapy.

TACE is a classic interventional treatment method for intermediate and advanced stage hepatocellular carcinoma. It precisely delivered chemotherapeutic drugs and embolic agents directly to the tumor through the hepatic artery, cutting off the tumor’s blood supply and increasing the local concentration of medication (3). This method has been shown to prolong survival rates in patients with intermediate-stage HCC. However, it also has its limitations. Due to incomplete embolization and the establishment of collateral blood vessels in the tumor, TACE often fails to achieve pathological necrosis. Additionally, postoperative ischemia and hypoxia in tumor tissues can increase the level of hypoxia-inducible factors in residual tumors and elevate the expression of vascular endothelial growth factor(VEGF), aggravating the disease and recurrence (4, 5).

In recent years, targeted therapy has made significant progress in the treatment of advanced liver cancer. Sorafenib, a multi-kinase inhibitor, suppresses tumor angiogenesis by targeting the VEGF signaling pathway, inhibiting tumor growth and metastasis. As the standard first-line treatment drug, it can significantly extend the overall survival of patients with advanced liver cancer (6, 7). However, the efficacy of sorafenib monotherapy remains limited, potentially leading drug resistance in some patients. Exploring combination therapy regimens has become a research hotspot. Studies have shown that sorafenib plus TACE may enhance the control of local tumor after TACE and mitigate hypoxia-driven metastasis (8). Thus, the combination of sorafenib with TACE has gained attention as a potential therapeutic strategy (9, 10).

With the incidence of HCC rising, optimizing treatment strategies for patients with intermediate to advanced liver cancer has become increasingly urgent. Studies have indicated that the combination of sorafenib and TACE may offer potential benefits in the treatment of HCC. However, there is a lack of systematic summary and evaluation, and inconsistencies in their results (11). To address this issue and provide valuable insights for clinical practice, we conducted a systematic review and meta-analysis focusing on the efficacy and safety of TACE plus sorafenib for the treatment of HCC (12, 13).

## 2 Methods

### 2.1 Literature search

During this systematic review, we adhered to the PRISMA guidelines (14). A comprehensive system retrieved online databases: PubMed, the Cochrane Library, Embase, Web of Science, the Chinese National Knowledge Infrastructure (CNKI), and the Wan-Fang Database in China without language restrictions on May 30, 2025. MeSH terms and free words related to “Hepatocellular Carcinoma”, “sorafenib” and “Therapeutic Chemoembolization” were used in this research. Details of search strategy were displayed in **Supplementary File S1**. Additionally, we manually screened the references of all identified articles for additional studies that might be relevant to this meta-analysis.

### 2.2 Inclusion and exclusion criteria

#### Inclusion criteria

1. Randomized controlled trials (RCTs).
2. Study patients were pathologically or clinically diagnosed with hepatocellular carcinoma, regardless of the kind of treatment that they have experienced before.
3. Two intervention arms (TACE plus sorafenib vs TACE alone) were compared in the study.
4. One of the following must be reported in study: partial response rate (PR), objective response rate (ORR), 12-month OS rates and adverse reactions.
5. There is no language restriction.

#### Exclusion criteria

1. Duplicate studies.
2. Non-RCTs.
3. Inconsistent experimental groupings.
4. The basic data was incomplete and the original data was unavailable.
5. Reviews, meta-analyses, non-clinical reports, case reports, conference summaries, opinions, editorials and letters.

### 2.3 Data extraction

Data was extracted independently by two investigators (Lixiu Yu and Yi Xiong) according to the inclusion and exclusion criteria. General information of the eligible studies including first author’s name, published year, study design, case of participants in the eligible studies, age, gender, Child-Pugh class. Efficacy outcome measures included PR, ORR, 12-month OS rates. Safety outcomes included typical adverse events (AEs) reported by patients. Disagreements between the investigators were resolved through discussion.

### 2.4 Quality assessment

The quality of the RCTs included in this meta-analysis was evaluated by Cochrane Collaboration’s risk of bias tool (15) from seven critical aspects: random sequence generation, allocation concealment, blinding of participants and personnel, blinding of outcome assessment, incomplete outcome data, selective reporting, other bias. Each aspect was judged as low, unclear, or high risk of bias. The quality assessment was conducted independently by two researchers (Yi Xiong and Lixiu Yu). Disagreements were resolved by discussion.

### 2.5 Data synthesis and analysis

Meta-analyses were conducted by using Review Manager 5.4. The pooled odds ratio (OR) and 95% confidence interval (CI) were employed to evaluate efficacy and safety. The I2 statistic was used to measure heterogeneity of the studies. If significant heterogeneity was detected with I² > 50%, the estimated outcomes of eligible studies were calculated using a random-effects model. Otherwise, a fixed-effects model was applied. And statistical test with p < 0.05 was considered significant.

## 3 Results

### 3.1 Study search and selection

**Figure 1:**
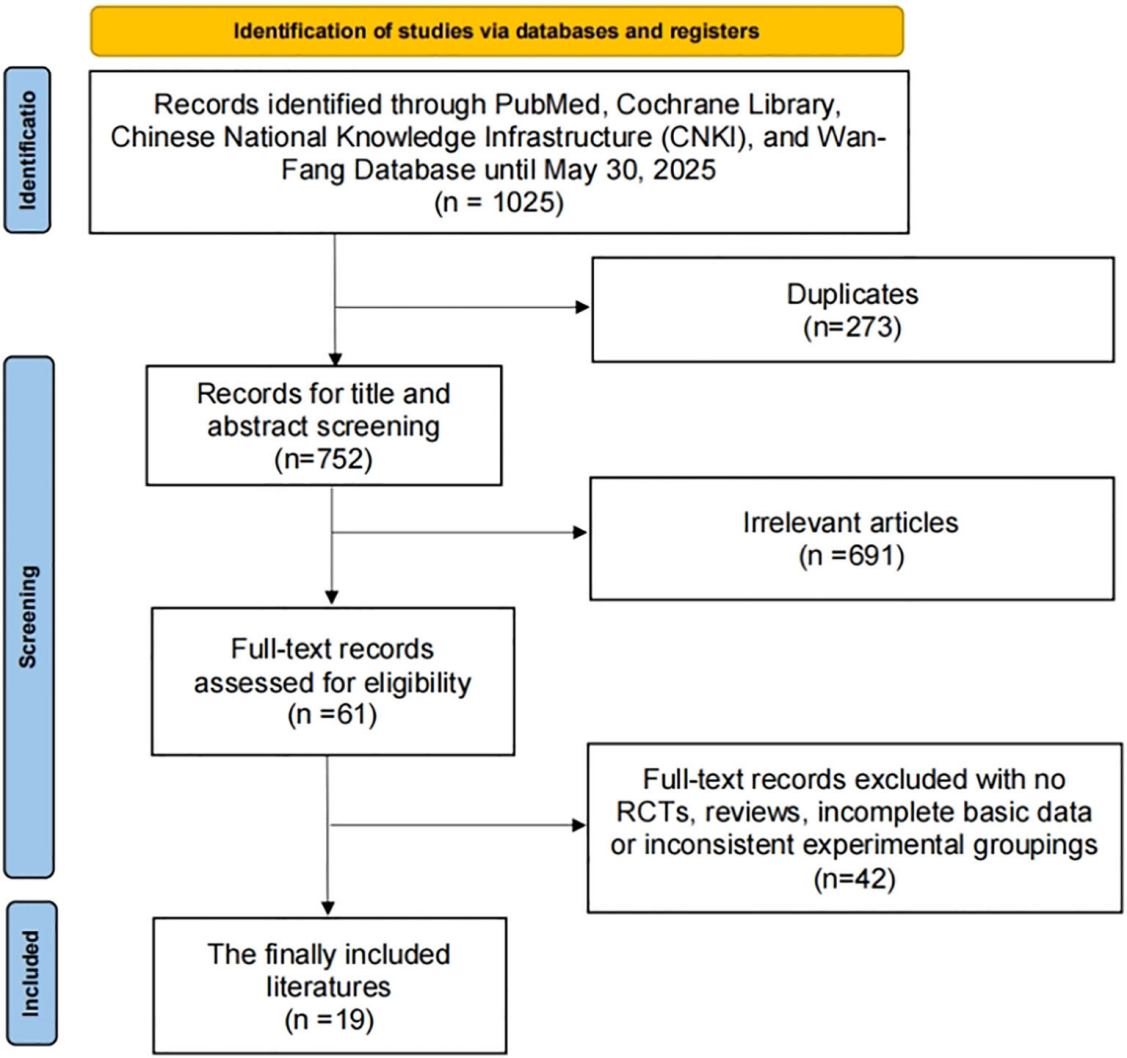
The process of selection of the eligible studies.

As depicted in **Figure 1**, the study selection process involved a comprehensive search of online databases and manual searches, yielding a total of 1025 articles. After eliminating 273 duplicate articles, further 691 articles were excluded based on their titles and abstracts. Upon full-text review and applying the inclusion criteria, 42 articles were excluded. Ultimately, 19 RCTs, comprising a total of 2,029 patients diagnosed with liver cancer, were included in this study (10, 16–33).

### 3.2 Study characteristics

A total of 19 RCTs were included in this meta-analysis, of these 17 studies (10, 16–19, 21–29, 31–33) reported Child-Pugh classification system. Majority of patients were classified as Child-Pugh class A. The baseline characteristics of patients were summarized in **Table 1**. Of the 2029 enrolled patients with HCC, 1,023 received sorafenib plus TACE, and1,006 were treated with TACE alone. The patients’ ages were between 45 and 75 years old. Sorafenib was administered orally at a dosage of 400 mg twice daily. The details of intervention characteristics and outcome measures of all the included trials were summarized in **Supplementary File S2**. All the trials (10, 16–33) used PR and ORR as outcome metrics, and eleven trials (10, 16, 19, 21, 22, 27, 29–33) reported 12-month OS rates.

**Table 1:** Baseline characteristic of eligible studies.

First author	Year	Study design	Group	Cases	Age (year)	Sex (male/female)	Child-Pugh class		
							A	B	C
Xinjian Wang (16)	2025	RCT	combination group	39	62.91 ± 3.75	25/14	29	10	0
			control group	39	63.18 ± 4.22	27/12	28	11	0
Yunyun Jie (17)	2024	RCT	combination group	37	60.77 ± 4.51	20/17	16	21	0
			control group	37	61.34 ± 4.45	22/15	18	19	0
Jiurong Zhu (18)	2024	RCT	combination group	24	54.26 ± 4.68	13/11	17	7	0
			control group	24	54.93 ± 5.54	14/10	16	8	0
Wenzhe Fan (19)	2024	RCT	combination group	81	54	72/9	81	0	0
			control group	81	58	79/2	81	0	0
Daolin Zeng (20)	2024	RCT	combination group	50	56.13 ± 4.72	27/23	NA	NA	NA
			control group	50	54.58 ± 5.37	29/21	NA	NA	NA
Xiaocen Wei (21)	2022	RCT	combination group	40	49.62± 2.14	23/17	24	16	0
			control group	40	49.24± 2.11	22/18	25	15	0
Quanguo Liu et al., 202022 (22)	2020	RCT	combination group	59	56.31 ± 9.87	37/22	43	16	0
			control group	59	58.11 ± 10.44	32/27	48	11	0
Haibo Zhu (23)	2020	RCT	combination group	23	58.50 ± 5.30	12/11	16	7	0
			control group	21	57.50 ± 5.10	11/10	15	6	0
Jingjie Pan (24)	2019	RCT	combination group	54	55.82 ± 13.04	36/18	35	19	0
			control group	53	54.96 ± 14.15	30/23	33	20	0
Masatoshi Kudo (10)	2019	RCT	combination group	80	72	63/17	79	1	0
			control group	76	73	55/21	71	5	0
Tim Meyer (25)	2017	RCT	combination group	157	65	139/18	145	5	0
			control group	156	68	138/18	148	3	0
Lei Li (26)	2017	RCT	combination group	38	53.70 ± 10.30	23/15	18	20	0
			control group	37	53.40 ± 10.50	22/15	17	20	0
Jiahang Xie (27)	2015	RCT	combination group	43	54.20 ± 7.10	34/9	30	13	0
			control group	40	53.9 ± 7.5	30/10	28	12	0
Yong Tan (28)	2015	RCT	combination group	29	52.3 ± 5.1	19/10	20	9	0
			control group	28	53.10 ± 5.30	17/11	19	9	0
Zhijian You (29)	2015	RCT	combination group	82	24-76	60/22	65	12	5
			control group	78	26-75	50/28	52	23	3
Rengui Zhou (30)	2014	RCT	combination group	48	71.90 ± 12.70	34/14	NA	NA	NA
			control group	52	67.90 ± 10.80	31/17	NA	NA	NA
Heng Sun (31)	2014	RCT	combination group	81	54.50 ± 7.90	68/13	70	11	0
			control group	81	53.90 ± 8.20	66/15	72	9	0
Siming Chen (32)	2012	RCT	combination group	28	68.2	20/8	15	13	0
			control group	28	68.2	17/11	16	12	0
Haiying Jiang (33)	2010	RCT	combination group	30	56	24/6	25	5	0
			control group	30	57	23/7	24	6	0

Ages are expressed as the median or mean ± standard deviation; NA: Not available.

### 3.3 Quality assessment

**Figure 2:**
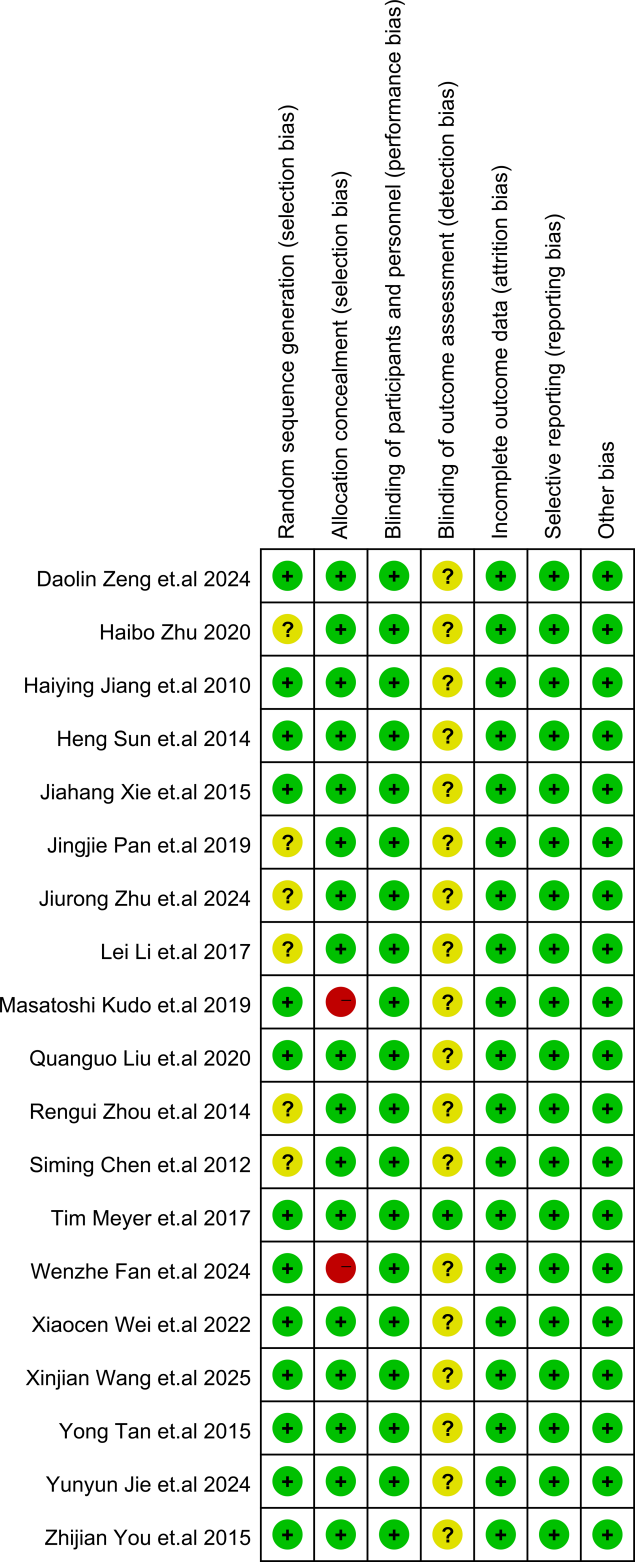
Risk of bias summary. In the illustration, green represents low risk, yellow represents unclear risk, and red represents high risk.

The details of study quality assessment were summarized in **Figure 2** and **Supplementary File S3**. The Cochrane Collaboration’s risk of bias tool was used to evaluate the quality of included studies. Overall, the included studies revealed a superior standard of methodological quality. Most studies presented a low risk of bias.

### 3.4 Meta-analysis results

#### 3.4.1 Efficacy of sorafenib combined with TACE

**Figure 3:**
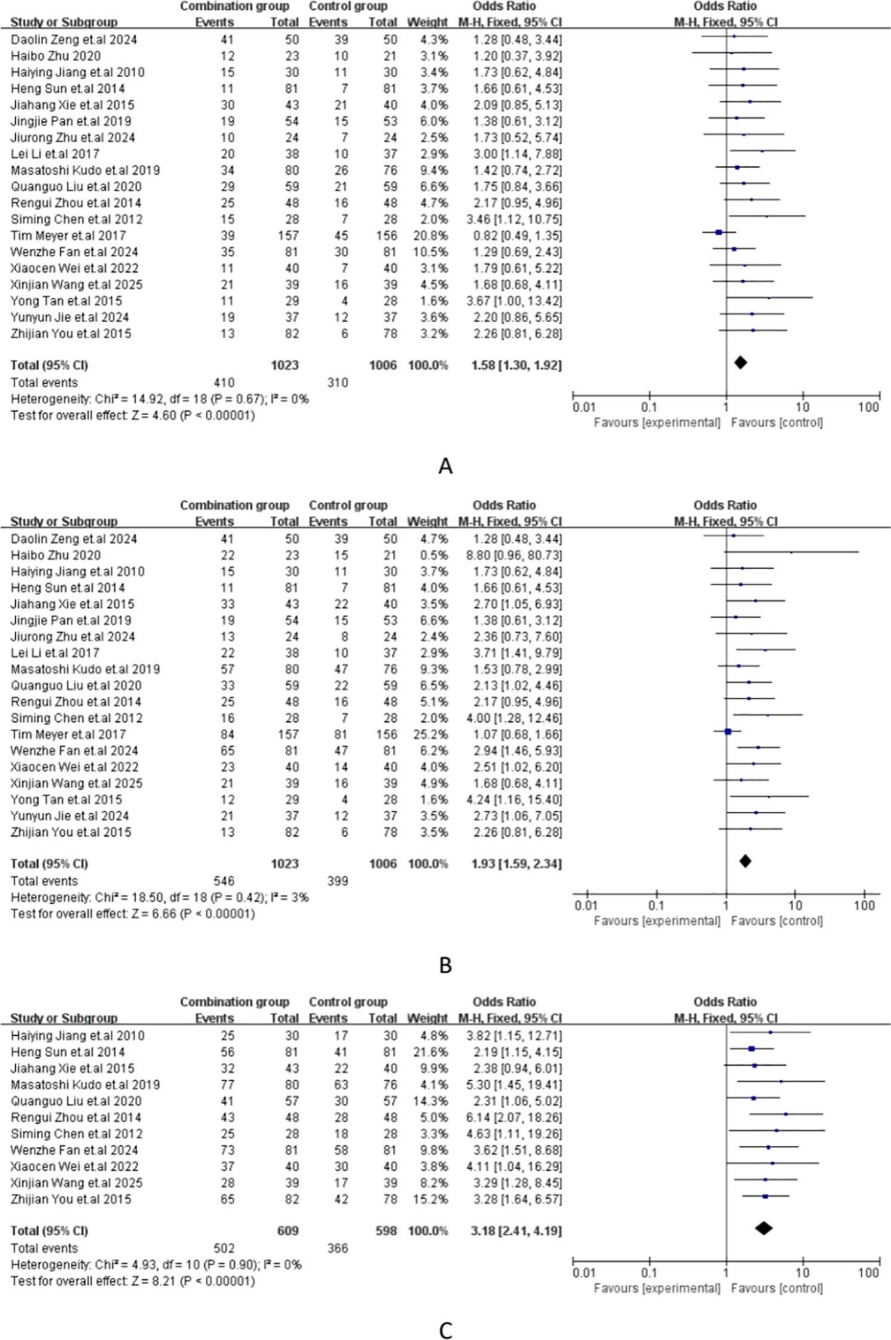
**(A)** Forest plot of partial response rate (PR); **(B)** Forest plot of objective response rate(ORR); **(C)** Forest plot of the 12-month OS rates.

**Figure 4:**
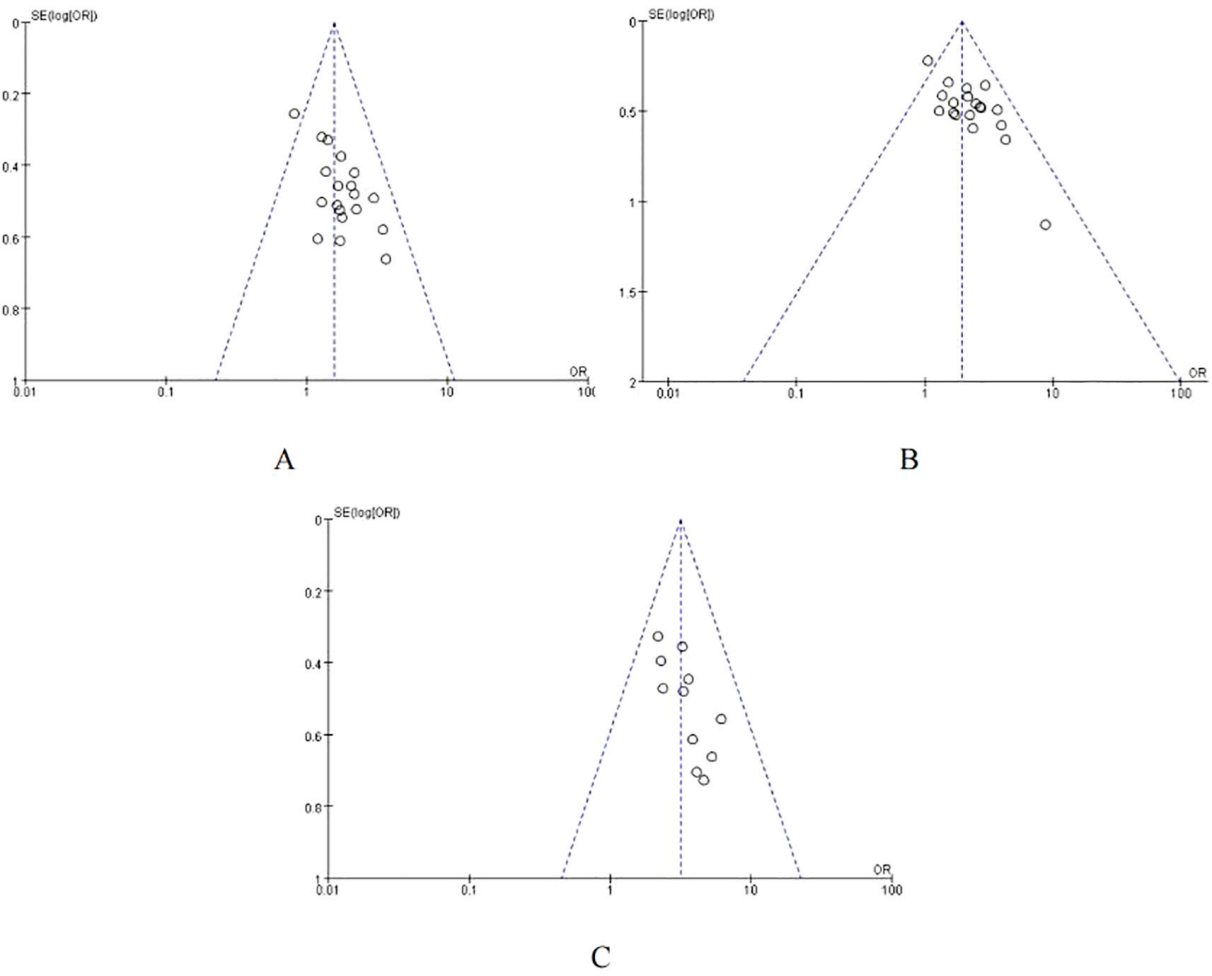
Funnel plot of ORR. **(A)** Funnel plot of PR; **(B)** Funnel plot of ORR; **(C)** Funnel plot of the 12-month OS rates.

The meta-analysis of all the included studies indicated that compared with TACE alone, Sorafenib combined with TACE had a favorable partial response rate (PR) with integrated OR = 1.58 (95% CI: 1.30–1.92), P = 0.67, I² = 0%. In addition, the result of objective response rate (ORR) with pooled OR = 1.93, 95%CI (1.59,2.34)] and 12-months OS rate with pooled OR = 3.18, 95%CI (2.41,4.19) demonstrated that TACE plus sorafenib had better clinical outcomes than TACE alone. The details of the efficacy and funnel plot were summarized in **Figures 3** and **4** respectively. One the other hand, the available data in **Table 2** suggested that both APF and VEGF levels decreased after treatment especially for TACE plus sorafenib, which further proved that the combination of TACE and sorafenib was an effective strategy.

**Table 2:** The responses of AFP and VEGF.

Studies	Group	Cases	AFP (ng/ml)		VEGF (pg/ml)	
			Before treatment	After treatment	Before treatment	After treatment
Xinjian Wang et al., 2025 (16)	treatment group	39	369.51 ± 50.32	92.93 ± 24.42	NA	NA
	control group	39	372.45 ± 51.45	121.72 ± 30.91	NA	NA
Yunyun Jie et al., 2024 (17)	treatment group	37	691.45 ± 130.57	133.67 ± 79.36	344.67 ± 64.56	271.46 ± 54.27
	control group	37	684.52 ± 123.66	231.44 ± 88.78	345.37 ± 66.47	228.67 ± 47.56
Jiurong Zhu et al., 2024 (18)	treatment group	24	222.26 ± 13.16	71.71 ± 6.31	NA	NA
	control group	24	219.43 ± 12.13	98.04 ± 9.01	NA	NA
Xiaocen Wei et al., 2022 (21)	treatment group	40	341.87 ± 34.21	158.39 ± 25.31	501.28 ± 35.42	278.53 ± 26.31
	control group	40	341.59 ± 34.19	187.42 ± 26.53	502.13 ± 35.49	316.53 ± 25.41
Quanguo Liu et al., 2020 (22)	treatment group	59	516.65 ± 104.31	292.86 ± 78.49	478.13 ± 70.92	254.28 ± 50.81
	control group	59	495.85 ± 110.93	361.18 ± 69.58	465.80 ± 84.69	337.47 ± 61.53
Lei Li et al., 2017 (26)	treatment group	38	738.2 ± 185.8	118.4± 46.7	331.0 ± 76.2	279.7 ± 57.6
	control group	37	720.6 ± 177.4	224.5 ± 102.1	342.8 ± 81.6	220.5 ± 40.8
Rengui Zhou et al., 2014 (30)	treatment group	48	176.9 ± 14.9	83.9 ± 10.2	NA	NA
	control group	52	172.7 ± 14.3	134.8 ± 13.5	NA	NA
Siming Chen et al., 2012 (32)	treatment group	28	182.36 ± 4.27	85.59 ± 7.2	NA	NA
	control group	28	169.83 ± 6.2	131.32 ± 12.8	NA	NA
Haiying Jiang et al., 2010 (33)	treatment group	30	NA	NA	261.78 ± 139.36	146.45 ± 120.23
	control group	30	NA	NA	262.67 ± 140.45	295.56 ± 127.27

NA: Not available.

#### 3.4.2 Subgroup analysis

**Figure 5:**
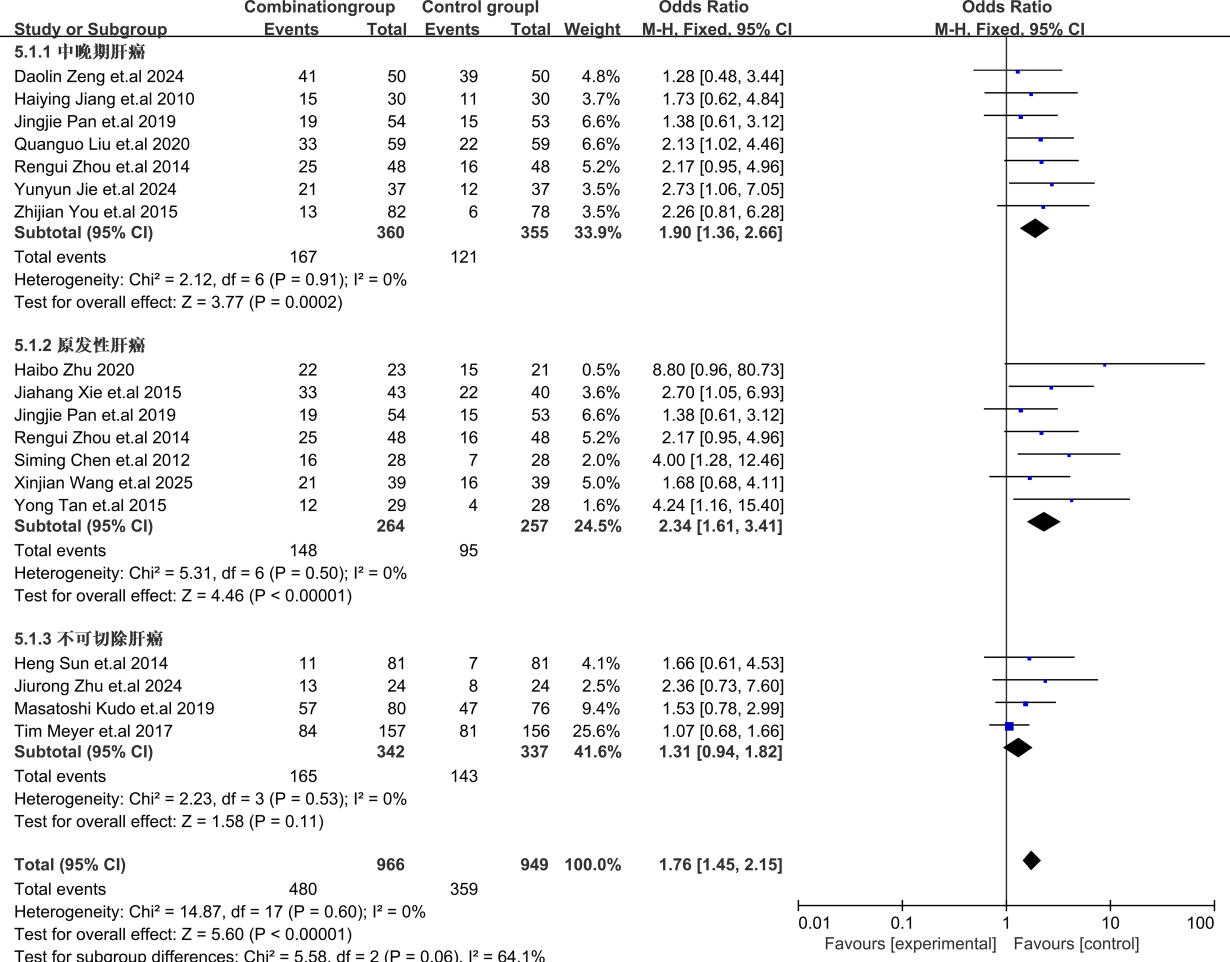
Forest plot of the subgroup analysis.

Subgroup analysis of different types of HCC showed that TACE plus sorafenib for patients with primary HCC provided the best benefits with OR = 2.34, 95% CI (1.61,3.41), I2 = 0%, P = 0.50, for intermediate-advanced HCC OR = 1.90, 95% CI (1.36,2.66), I2 = 0%, P = 0.91], and for unresectable HCC OR = 1.31, 95% CI (0.94,1.81), I2 = 0%, P = 0.53. These results indicated that sorafenib combined with TACE showed a statistically significant difference in efficacy compared with TACE alone for the treatment of intermediate-advanced and primary HCC. However, for the treatment of unresectable HCC, there was no statistically significant difference in efficacy. The subgroup analysis details were presented in **Figure 5**.

#### 3.4.3 Safety evaluation

**Figure 6:**
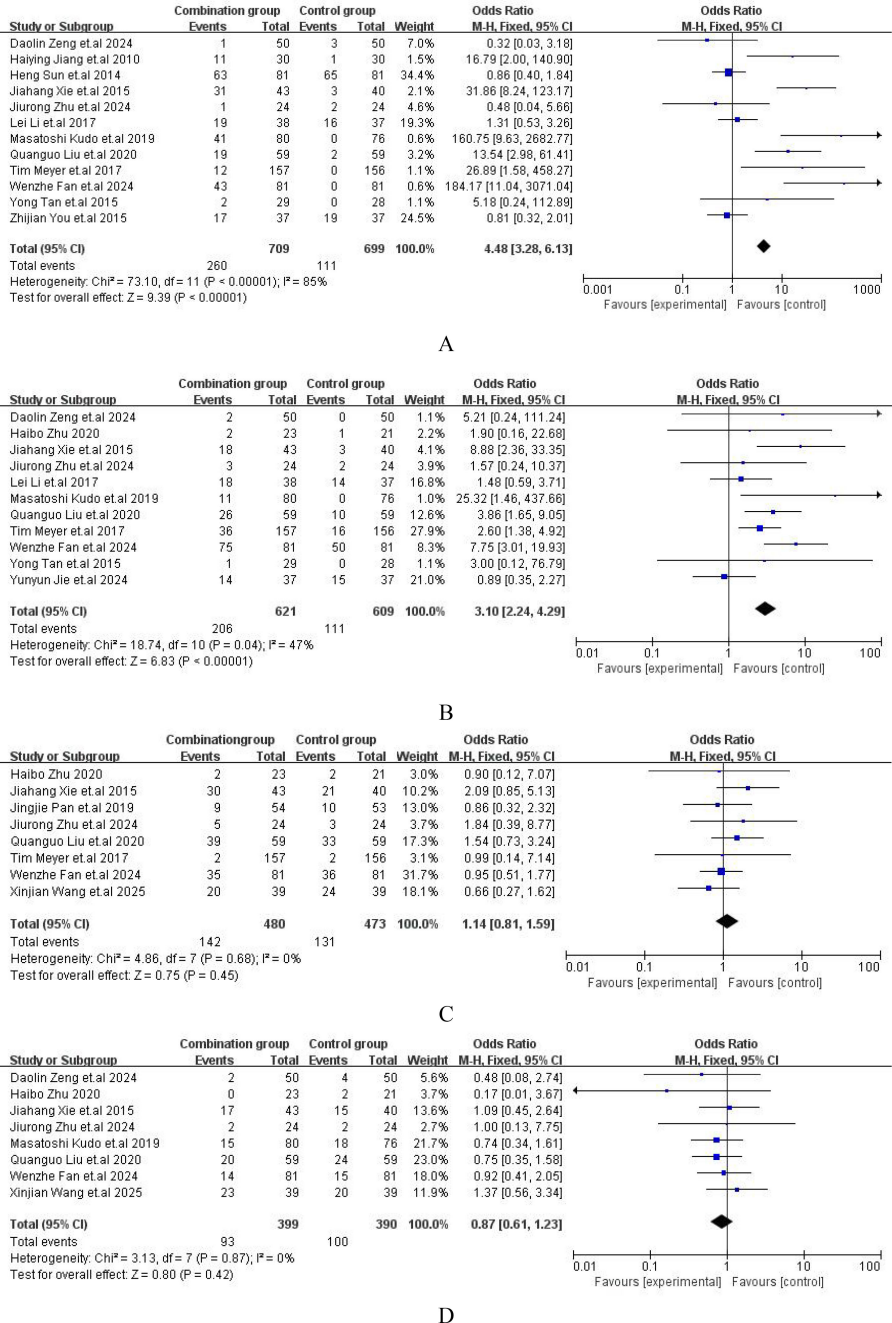
Forest plot of safety analysis. **(A)** Hand and foot skin reaction; **(B)** Diarrhea or abdominal pain; **(C)** Nausea or vomit; **(D)** Fever.

The major adverse events (AEs) from all included studies were presented in **Supplementary File S4**. Compared with TACE alone, the incidence of hand-foot skin reaction [OR = 4.48, 95%CI (3.28,6.13), P < 0.00001] (**Figure 6A**) and abdominal pain or diarrhea [OR = 3.10, 95%CI (2.24,4.29), P = 0.04] (**Figure 6B**) were both higher than those with TACE plus sorafenib. However, the incidence of nausea or vomiting [OR = 1.14, 95%CI (0.81,1.59), P = 0.68] (**Figure 6C**) or fever [OR = 0.87, 95%CI (0.61,1.23), P = 0.87] (**Figure 6D**) between the two groups was no significant difference. The comprehensive details of the safety were outlined in **Figure 6**.

## 4 Discussion

In the present study, we systematically evaluated the efficacy and safety of sorafenib in combination with TACE versus TACE alone on the treatment of HCC, providing a definitive systematic review with updated data. A total of 19 randomized controlled trials (10, 16–33), encompassing 2029 patients, were identified and included in this meta-analysis. The results indicated that compared with TACE alone, the combination of sorafenib and TACE significantly improved patient prognosis. Our findings further confirmed the combination of TACE and sorafenib significantly improved the clinical outcomes for the treatment of HCC.

In terms of efficacy, the combined therapy demonstrated a significant advantage over TACE alone in PR, ORR and the 12-month OS rates. Notably, the combination regimen yielded a substantial clinical benefit with an OR = 1.93 for ORR, which was consistent with the report by Chen et al. (34) (OR = 2.19). Sorafenib is a multi-kinase inhibitor that inhibits the activity of vascular endothelial growth factor receptor (VEGFR) and the platelet-derived growth factor receptor (PDGFR) and reduce the formation of new blood vessels in HCC tumors. It can also reduce the number and activity of immune-suppressive cells (such as regulatory T cells and myeloid-derived suppressor cells), and strengthen anti-tumor immunity (35). TACE works by embolizing the feeding arteries to the tumor, and reducing its blood supply, leading to tumor shrinkage and has an improved prognosis for HCC patients. Studies proved that TACE plus sorafenib had significantly enhanced the efficacy for HCC patients (36–39).

Subgroup analysis revealed that the efficacy of combination therapy was superior to TACE alone in patients with intermediate-advanced HCC and primary HCC. However, the benefit for unresectable HCC patients was not significant with OR = 1.31(0.94,1.82). There were some discrepancies between these findings and the TACTICS trial (10) which conducted by several domestic centers and reported significant clinical benefits of sorafenib plus TACE in unresectable HCC. The variation for this may be related to sample size and differences in sorafenib regimens. Increasing sample size tends to reduce the error. TACTICS trial only included 156 patients with unresectable HCC, while our work included 679 patients with this subtype. Additionally, the regimens for sorafenib may have some impacts on the outcomes. Among TACTICS trial sorafenib was initiated 14–21 days before TACE at a dose of 400 mg once daily, while this systematical study included both sorafenib initiated before TACE and after TACE regimens. Subgroup analysis indicated that for sorafenib initiated before TACE OR = 1.68(1.25, 2.27), while for sorafenib initiated after TACE OR = 2.32(1.74, 3.11). It still needs further confirmation by more high-quality RCTs with large samples and reliable design.

The present work indicated that there was no significant difference between TACE plus sorafenib group and TACE alone group in aspects of nausea, vomiting and fever. However, the safety results also suggested that compared with TACE alone, the combination of TACE and sorafenib significantly increased the risk of hand-foot skin reaction (HFSR) [OR = 4.48,95%CI (3.28,6.13)], abdominal pain and diarrhea [OR = 3.10,95%CI (2.24,4.29)]. The increased risk of these may relate to the pharmacological properties of sorafenib. It inhibited multiple intracellular signaling pathways in liver carcinoma cells. It can inhibit angiogenesis in tumor tissues and block cancer cell proliferation by inhibiting kinase activities, such as those of c-Raf, VEGFR2, VEGFR3, which can affect the function of the skin and blood vessels leading to HFSR (42). Sorafenib can also lead to a decrease in intestinal mucosal blood supply and repair ability, thereby causing abdominal pain and diarrhea (40). Prioritizing the management of treatment-related toxicities is essential to maintain patient quality of life and ensure treatment adherence (25). It is crucial to find potential ways to reduce adverse reactions, such as providing skin care guidance, adjusting drug doses, and administering antidiarrheal and rehydration therapies.

This study boasts several notable strengths: firstly, our work systematically analyzed 19 RCTs, which had been significantly enhanced in terms of credibility. Secondly, by applying rigorous literature screening criteria and utilizing the Cochrane risk-of-bias tool for quality assessment, the reliability of our research findings is significantly enhanced. Thirdly, in this study, we conducted subgroup analyses to explore differential treatment responses among various patient and comprehensively evaluated the efficacy and safety of TACE plus sorafenib, which indicated that further research needed to fully elucidate the mechanisms behind these observations.

There were some limitations to this study that warranted attention. Firstly, although a quality assessment was conducted, the majority of the trials included exhibited uncertain risks of bias. These uncertainties could potentially impact the accuracy of our study results (41). Secondly, there were some variations in the studies whose data have been complied together not only with different patient characteristics but geographic regions, and most of the articles did not stratify the severity of adverse events. We were also unable to adjust for confounders such as age, sex and medical duration of HCC. In addition, HCC is a highly heterogeneous disease in treatment responses among patients. Other factors related to tumor biology (such as genetic mutation profiles and tumor microenvironment), host factors (such as liver function status and overall health condition), and environmental and lifestyle factors also influence disease progression and prognosis. Due to limited data, information on other relevant factors were not extracted, which may cause some bias in our results. In light of these limitations, future research should conduct more large-scale, multicenter RCTs to minimize individual variability and sampling bias.

## 5 Conclusion

The combination of TACE plus sorafenib offers therapeutic benefits in the treatment of HCC and may be a choice of treatment in patients with unresectable HCC.

## Data Availability

The original contributions presented in the study are included in the article/**Supplementary Material**. Further inquiries can be directed to the corresponding author.

## References

[1] Rumgay H Arnold M Ferlay J Lesi O Cabasag CJ Vignat J . Global burden of primary liver cancer in 2020 and predictions to 2040. J Hepatol. (2022) 77:1598–606. doi: 10.1016/j.jhep.2022.08.021, PMID: 36208844 PMC9670241

[2] Alawyia B Constantinou C . Hepatocellular carcinoma: a narrative review on current knowledge and future prospects. Curr Treat Options Oncol. (2023) 24:711–24. doi: 10.1007/s11864-023-01098-9, PMID: 37103744

[3] Chang Y Jeong SW Young Jang J Jae Kim Y . Recent updates of transarterial chemoembolilzation in hepatocellular carcinoma. Int J Mol Sci. (2020) 21:8165. doi: 10.3390/ijms21218165, PMID: 33142892 PMC7662786

[4] Zhou B Wang J Yan Z . Ginsenoside Rg3 attenuates hepatoma VEGF overexpression after hepatic artery embolization in an orthotopic transplantation hepatocellular carcinoma rat model. Onco Targets Ther. (2014) 7:1945–54. doi: 10.2147/OTT.S69830, PMID: 25364265 PMC4211851

[5] Tao Z Chen B Tan X Zhao Y Wang L Zhu T . Coexpression of VEGF and angiopoietin-1 promotes angiogenesis and cardiomyocyte proliferation reduces apoptosis in porcine myocardial infarction (MI) heart. Proc Natl Acad Sci U S A. (2011) 108:2064–9. doi: 10.1073/pnas.1018925108, PMID: 21245320 PMC3033313

[6] European Association for the Study of the Liver . EASL Clinical Practice Guidelines: Management of hepatocellular carcinoma. J Hepatol. (2018) 69:182–236. doi: 10.1016/j.jhep.2018.03.019, PMID: 29628281

[7] Kelley RK Rimassa L Cheng AL Kaseb A Qin S Zhu AX . Cabozantinib plus atezolizumab versus sorafenib for advanced hepatocellular carcinoma (COSMIC-312): a multicentre, open-label, randomised, phase 3 trial. Lancet Oncol. (2022) 23:995–1008. doi: 10.1016/S1470-2045(22)00326-6, PMID: 35798016

[8] Kimura Y Kaneko R Yano Y Kamada K Ikehara T Nagai H . The prognosis of hepatocellular carcinoma treated with sorafenib in combination with TACE. Asian Pac J Cancer Prev. (2020) 21:1797–805. doi: 10.31557/APJCP.2020.21.6.1797, PMID: 32592380 PMC7568902

[9] Pawlik TM Reyes DK Cosgrove D Kamel IR Bhagat N Geschwind JF . Phase II trial of sorafenib combined with concurrent transarterial chemoembolization with drug-eluting beads for hepatocellular carcinoma. J Clin Oncol. (2011) 29:3960–7. doi: 10.1200/JCO.2011.37.1021, PMID: 21911714 PMC4829081

[10] Kudo M Ueshima K Ikeda M Torimura T Tanabe N Aikata H . Randomised, multicentre prospective trial of transarterial chemoembolisation (TACE) plus sorafenib as compared with TACE alone in patients with hepatocellular carcinoma: TACTICS trial. Gut. (2020) 69:1492–501. doi: 10.1136/gutjnl-2019-318934, PMID: 31801872 PMC7398460

[11] Zhang L Hu P Chen X Bie P . Transarterial chemoembolization (TACE) plus sorafenib versus TACE for intermediate or advanced stage hepatocellular carcinoma: a meta-analysis. PloS One. (2014) 9:e100305. doi: 10.1371/journal.pone.0100305, PMID: 24945380 PMC4063775

[12] Leal CRG Magalhães C Barbosa D Aquino D Carvalho B Balbi E . Survival and tolerance to sorafenib in Child-Pugh B patients with hepatocellular carcinoma: a prospective study. Invest New Drugs. (2018) 36:911–18. doi: 10.1007/s10637-018-0621-x, PMID: 29948358

[13] Pan S Zheng J Shi C . Analysis and prediction of the efficacy and influencing factors of camrelizumab combined with TACE and sorafenib in the treatment of advanced hepatocellular carcinoma. J Cancer Res Clin Oncol. (2023) 149:12479–87. doi: 10.1007/s00432-023-05050-0, PMID: 37450029 PMC11796889

[14] Page MJ McKenzie JE Bossuyt PM Boutron I Hoffmann TC Mulrow CD . The PRISMA 2020 statement: An updated guideline for reporting systematic reviews. Int J Surg (Lond Engl). (2021) 88:105906. doi: 10.1016/j.ijsu.2021.105906, PMID: 33789826

[15] Higgins JP Altman DG Gøtzsche PC Jüni P Moher D Oxman AD . Cochrane Collaboration’s tool for assessing risk of bias in randomised trials. BMJ. (2011) 343:d5928. doi: 10.1136/bmj.d5928, PMID: 22008217 PMC3196245

[16] Wang X Wang P He Y Men C . Effects of Sorafenib combined with transcatheter arterial chemoembolization in treatment of patients with primary liver cancer. Med J Chin People’s Health. (2025) 37:44–6. doi: 10.3969/j.issn.1672-0369.2025.01.014

[17] Jie Y Yu R Zhang F Fan X Liu H . Clinical effects of sorafenib combined with TACE in the treatment of hepatocellular carcinoma and its impact on serum factor levels in patients. Chin J Clin Rational Drug Use. (2024) 17:12–5. doi: 10.15887/j.cnki.13-1389/r.2024.25.004

[18] Zhu J Yue K Ye Y Wu Y . Efficacy analysis of sorafenib tosylate with TACE in the treatment of unresectable localized liver cancer patients. Pract J Cancer. (2024) 39:648–651+677. doi: 10.3969/j.issn.1001-5930.2024.04.032

[19] Fan W Zhu B Chen S Wu Y Zhao X Qiao L . Survival in patients with recurrent intermediate-stage hepatocellular carcinoma: sorafenib plus TACE vs TACE alone randomized clinical trial. JAMA Oncol. (2024) 10:1047–54. doi: 10.1001/jamaoncol.2024.1831, PMID: 38900435 PMC11190833

[20] Zeng D Yu C Chen S Zou L Chen J Xu L . Assessment of disease control rate and safety of sorafenib in targeted therapy for advanced liver cancer. World J Surg Oncol. (2024) 22:93. doi: 10.1186/s12957-024-03364-y, PMID: 38605359 PMC11010384

[21] Wei X . Effects of sorafenib combined with TACE on immune function and alpha-fetoprotein levels in patients with hepatocellular carcinoma. Med Innovation China. (2022) 19:56–60. doi: 10.3969/j.issn.1674-4985.2022.21.014

[22] Liu Q Dai Y . Sorafenib combined with transarterial chemoembolization prolongs survival of patients with advanced hepatocellular carcinoma. J BUON. (2020) 25:945–51. Available online at: https://pubmed.ncbi.nlm.nih.gov/32521890/ 32521890

[23] Zhu H . Observation on the efficacy and safety of thalafinil combined with transcatheter arterial chemoembolization (TACE) in the treatment of primary liver cancer. Health Guide. (2020) 27:7–8. Available online at https://d.wanfangdata.com.cn/periodical/ysbjzn-x202027006

[24] Pan J Liu T-y Huang J Liu W . Clinical observation of transarterial chemoembolization combined with corafinil and compound berberine in treating patients with advanced liver cancer. J Oncol Chin Med. (2019) 1:38–44. doi: 10.19811/j.cnki.issn2096-6628.2019.04.009

[25] Meyer T Fox R Ma YT Ross PJ James MW Sturgess R . Sorafenib in combination with transarterial chemoembolisation in patients with unresectable hepatocellular carcinoma (TACE 2): a randomised placebo-controlled, double-blind, phase 3 trial. Lancet Gastroenterol Hepatol. (2017) 2:565–75. doi: 10.1016/S2468-1253(17)30156-5, PMID: 28648803

[26] Li L Zhong M Song H . Effects of sorafenib combined with TACE on serum cytokine expression in patients with hepatocellular carcinoma. Modern Digest Intervent. (2017) 22:482–4. doi: 10.3969/j.issn.1672-2159.2017.04.010

[27] Xie J Wang J . Analysis of efficacy and complications of sorafenib combined with transcatheter arterial chemoembolization (TACE) for advanced hepatocellular carcinoma. J Cancer Control Treat. (2015) 28:152–4. doi: 10.3969/j.issn.1674-0904.2015.03.010

[28] Tan Y Zhang T Peng J Zhao Y . Curative effect of sorafenib combined with transcatheter arterial chemoembolization in treatment of large primary liver cancer. Med J Natl Defending Forces Southwest China. (2015) 25:386–9. doi: 10.3969/j.issn.004-0188.2015.04.014

[29] You Z Zheng R Hu X Lin S Ye Z . Curative efficacy of transcatheter arterial chemoembolization combined with sorafenib for unresectable hepatocellular carcinoma. Chin J Clin Oncol Rehabil. (2015) 22:1212–4. doi: 10.13455/j.cnki.cjcor.2015.10.19

[30] Zhou R Zhou X Li X Xia Y Song P Guo X . Clinical effects of sorafenib combined with transcatheter arterial chemoembolization on the treatment of advanced primary hepatocellular. Prog Modern Biomed. (2014) 14:2494–6. doi: 10.13241/j.cnki.pmb.2014.13.025

[31] Sun H Han W . Analysis of curative effect of transcatheter arterial chemoembolization combined with sorafenib for not operation resection of hepatocellular carcinoma. Chin J Gastroenterol Hepatol. (2014) 23:486–8. doi: 10.3969/j.issn.1006-5709.2014.05.002

[32] Chen S Wang Y Xie H . Clinical observation of sorafenib combined with transcatheter arterial chem oembolization for treating senile primary carcinoma of the liver. China Joumal Modem Med. (2012) 22:71–3. doi: 10.3969/j.issn.1005-8982.2012.25.018

[33] Jiang H Xie X . Sorafenib combined with transcatheter arterial chemoembolization in the treatment of advanced hepatocellular carcinoma. Hainan Med J. (2010) 21:6–9. doi: 10.3969/j.issn.1003-6350.2010.23.003

[34] Chen A Li S Yao Z Hu J Cao J Topatana W . Adjuvant transarterial chemoembolization to sorafenib in unresectable hepatocellular carcinoma: A meta-analysis. J Gastroenterol Hepatol. (2021) 36:302–10. doi: 10.1111/jgh.15180, PMID: 32652685

[35] Llovet JM Ricci S Mazzaferro V Hilgard P Gane E Blanc JF . Sorafenib in advanced hepatocellular carcinoma. N Engl J Med. (2008) 359:378–90. doi: 10.1056/NEJMoa0708857, PMID: 18650514

[36] Chao Y Chung YH Han G Yoon JH Yang J Wang J . The combination of transcatheter arterial chemoembolization and sorafenib is well tolerated and effective in Asian patients with hepatocellular carcinoma: final results of the START trial. Int J Cancer. (2015) 136:1458–67. doi: 10.1002/ijc.29126, PMID: 25099027

[37] Li Y Zheng YB Zhao W Liu B Hu BS He X . Sorafenib in combination with transarterial chemoembolization and radiofrequency ablation in the treatment for unresectable hepatocellular carcinoma. Med Oncol. (2013) 30:730. doi: 10.1007/s12032-013-0730-5, PMID: 24048774

[38] Liang HY Lu LG Hu BS Li Y Shao PJ . Effects of sorafenib combined with chemoembolization and radiofrequency ablation for large, unresectable hepatocellular carcinomas. Chin Med J (Engl). (2013) 126:4270–6. doi: 10.3760/cma.j.issn.0366-6999.20120478, PMID: 24238511

[39] Yang HJ Ye B Liao JX Lei L Chen K . Sorafenib plus transarterial chemoembolization vs sorafenib alone for patients with advanced hepatocellular carcinoma: A systematic review and meta-analysis. World J Hepatol. (2024) 16:91–102. doi: 10.4254/wjh.v16.i1.91, PMID: 38313249 PMC10835483

[40] Mir O Coriat R Boudou-Rouquette P Durand JP Goldwasser F . Sorafenib-induced diarrhea and hypophosphatemia: mechanisms and therapeutic implications. Ann Oncol. (2012) 23:280–1. doi: 10.1093/annonc/mdr525, PMID: 22056851

[41] Sterne JAC Savović J Page MJ Elbers RG Blencowe NS Boutron I . RoB 2: a revised tool for assessing risk of bias in randomised trials. BMJ. (2019) 366:l4898. doi: 10.1136/bmj.l4898, PMID: 31462531

[42] Ai L Xu Z Yang B He Q Luo P . Sorafenib-associated hand-foot skin reaction: practical advice on diagnosis, mechanism, prevention, and management. Expert Rev Clin Pharmacol. (2019) 12:1121–7. doi: 10.1080/17512433.2019.1689122, PMID: 31679411

