# Supplementary material for: Efficacy and safety of sorafenib combined with transarterial chemoembolization in the treatment of hepatocellular carcinoma: a meta-analysis of randomized controlled trials
Source: Front Oncol. 2025 Nov 10;15:1640879. doi: 10.3389/fonc.2025.1640879 (PMC12640826; doi:10.3389/fonc.2025.1640879)
Supplement: Supplementary file 1 [file DataSheet1.doc]

**Supplemental Files S1.** Retrieval strategy

**PubMed: 58 records**

#1:"Carcinoma, Hepatocellular"[Mesh]

#2:(((((((((((((((((Carcinoma, Hepatocellular[Title/Abstract]) OR (Carcinomas, Hepatocellular[Title/Abstract])) OR (Hepatocellular Carcinomas[Title/Abstract])) OR (Hepatocellular Carcinoma[Title/Abstract])) OR (Hepatoma;Hepatomas[Title/Abstract])) OR (Liver Cancer, Adult[Title/Abstract])) OR (Adult Liver Cancer[Title/Abstract])) OR (Adult Liver Cancers[Title/Abstract])) OR (Cancer, Adult Liver[Title/Abstract])) OR (Cancers, Adult Liver[Title/Abstract])) OR (Liver Cancers, Adult[Title/Abstract])) OR (Liver Cell Carcinoma[Title/Abstract])) OR (Carcinoma, Liver Cell[Title/Abstract])) OR (Carcinomas, Liver Cell[Title/Abstract])) OR (Cell Carcinoma, Liver[Title/Abstract])) OR (Cell Carcinomas, Liver[Title/Abstract])) OR (Liver Cell Carcinomas[Title/Abstract])) OR (Liver Cell Carcinoma, Adul[Title/Abstract])

#3:("Carcinoma, Hepatocellular"[Mesh]) OR ((((((((((((((((((Carcinoma, Hepatocellular[Title/Abstract]) OR (Carcinomas, Hepatocellular[Title/Abstract])) OR (Hepatocellular Carcinomas[Title/Abstract])) OR (Hepatocellular Carcinoma[Title/Abstract])) OR (Hepatoma;Hepatomas[Title/Abstract])) OR (Liver Cancer, Adult[Title/Abstract])) OR (Adult Liver Cancer[Title/Abstract])) OR (Adult Liver Cancers[Title/Abstract])) OR (Cancer, Adult Liver[Title/Abstract])) OR (Cancers, Adult Liver[Title/Abstract])) OR (Liver Cancers, Adult[Title/Abstract])) OR (Liver Cell Carcinoma[Title/Abstract])) OR (Carcinoma, Liver Cell[Title/Abstract])) OR (Carcinomas, Liver Cell[Title/Abstract])) OR (Cell Carcinoma, Liver[Title/Abstract])) OR (Cell Carcinomas, Liver[Title/Abstract])) OR (Liver Cell Carcinomas[Title/Abstract])) OR (Liver Cell Carcinoma, Adul[Title/Abstract]))

#4:"Sorafenib"[Mesh]

#5:(((((((((Sorafenib[Title/Abstract]) OR (BAY 43-9006[Title/Abstract])) OR (BAY 439006[Title/Abstract])) OR (BAY 43 9006[Title/Abstract])) OR (Sorafenib Tosylate[Title/Abstract])) OR (4-(4-(3-(4-Chloro-3-trifluoromethylphenyl)ureido)phenoxy)pyridine-2-carboxylic acid methyamide-4-methylbenzenesulfonate[Title/Abstract])) OR (Nexavar[Title/Abstract])) OR (BAY 545 9085[Title/Abstract])) OR (Sorafenib N-Oxide[Title/Abstract])) OR (Sorafenib N Oxide[Title/Abstract])

#6: ("Sorafenib"[Mesh]) OR ((((((((((Sorafenib[Title/Abstract]) OR (BAY 43-9006[Title/Abstract])) OR (BAY 439006[Title/Abstract])) OR (BAY 43 9006[Title/Abstract])) OR (Sorafenib Tosylate[Title/Abstract])) OR (4-(4-(3-(4-Chloro-3-trifluoromethylphenyl)ureido)phenoxy)pyridine-2-carboxylic acid methyamide-4-methylbenzenesulfonate[Title/Abstract])) OR (Nexavar[Title/Abstract])) OR (BAY 545 9085[Title/Abstract])) OR (Sorafenib N-Oxide[Title/Abstract])) OR (Sorafenib N Oxide[Title/Abstract]))

#7:"Chemoembolization, Therapeutic"[Mesh]

#8:(((Chemoembolization, Therapeutic[Title/Abstract]) OR (Therapeutic Chemoembolization[Title/Abstract])) OR (Chemoembolizations, Therapeutic[Title/Abstract])) OR (Therapeutic Chemoembolizations[Title/Abstract])

#9:("Chemoembolization, Therapeutic"[Mesh]) OR ((((Chemoembolization, Therapeutic[Title/Abstract]) OR (Therapeutic Chemoembolization[Title/Abstract])) OR (Chemoembolizations, Therapeutic[Title/Abstract])) OR (Therapeutic Chemoembolizations[Title/Abstract]))

#10:randomized controlled trial

#11:(((("Carcinoma, Hepatocellular"[Mesh]) OR ((((((((((((((((((Carcinoma, Hepatocellular[Title/Abstract]) OR (Carcinomas, Hepatocellular[Title/Abstract])) OR (Hepatocellular Carcinomas[Title/Abstract])) OR (Hepatocellular Carcinoma[Title/Abstract])) OR (Hepatoma;Hepatomas[Title/Abstract])) OR (Liver Cancer, Adult[Title/Abstract])) OR (Adult Liver Cancer[Title/Abstract])) OR (Adult Liver Cancers[Title/Abstract])) OR (Cancer, Adult Liver[Title/Abstract])) OR (Cancers, Adult Liver[Title/Abstract])) OR (Liver Cancers, Adult[Title/Abstract])) OR (Liver Cell Carcinoma[Title/Abstract])) OR (Carcinoma, Liver Cell[Title/Abstract])) OR (Carcinomas, Liver Cell[Title/Abstract])) OR (Cell Carcinoma, Liver[Title/Abstract])) OR (Cell Carcinomas, Liver[Title/Abstract])) OR (Liver Cell Carcinomas[Title/Abstract])) OR (Liver Cell Carcinoma, Adul[Title/Abstract]))) AND (("Sorafenib"[Mesh]) OR ((((((((((Sorafenib[Title/Abstract]) OR (BAY 43-9006[Title/Abstract])) OR (BAY 439006[Title/Abstract])) OR (BAY 43 9006[Title/Abstract])) OR (Sorafenib Tosylate[Title/Abstract])) OR (4-(4-(3-(4-Chloro-3-trifluoromethylphenyl)ureido)phenoxy)pyridine-2-carboxylic acid methyamide-4-methylbenzenesulfonate[Title/Abstract])) OR (Nexavar[Title/Abstract])) OR (BAY 545 9085[Title/Abstract])) OR (Sorafenib N-Oxide[Title/Abstract])) OR (Sorafenib N Oxide[Title/Abstract])))) AND (("Chemoembolization, Therapeutic"[Mesh]) OR ((((Chemoembolization, Therapeutic[Title/Abstract]) OR (Therapeutic Chemoembolization[Title/Abstract])) OR (Chemoembolizations, Therapeutic[Title/Abstract])) OR (Therapeutic Chemoembolizations[Title/Abstract])))) AND (randomized controlled trial)

**Embase: 207 records**

#1：'liver cell carcinoma'/exp

#2：'carcinoma in the liver':ab,ti OR 'carcinoma of the liver':ab,ti OR 'carcinoma, hepatic cell':ab,ti OR 'carcinoma, hepatocellular':ab,ti OR 'carcinoma, liver':ab,ti OR 'carcinoma, liver cell':ab,ti OR 'hepatic carcinoma':ab,ti OR 'hepatic cell carcinoma':ab,ti OR 'hepato-carcinoma':ab,ti OR 'hepato-cellular carcinoma':ab,ti OR 'hepatocarcinoma':ab,ti OR 'hepatocellular carcinoma':ab,ti OR 'hepatocellular carcinomata':ab,ti OR 'hepatocyte carcinoma':ab,ti OR 'hepatocytic carcinoma':ab,ti OR 'hepatoma':ab,ti OR 'hepatomata':ab,ti OR 'hepatomatous':ab,ti OR 'liver carcinoma':ab,ti OR 'liver carcinoma rupture':ab,ti OR 'malignant hepatoma':ab,ti OR 'primary liver carcinoma':ab,ti OR 'liver cell carcinoma':ab,ti

#3：#1 OR #2

#4:'sorafenib'/exp

#5:sorafenib:ab,ti OR ('4 [4 [3 [4 chloro 3':ab,ti AND trifluoromethyl:ab,ti AND 'phenyl] ureido] phenoxy] n methyl 2 pyridinecarboxamide':ab,ti) OR 'bay 43 9006':ab,ti OR 'bay 43-9006':ab,ti OR 'bay 439006':ab,ti OR 'bay 54 9085':ab,ti OR 'bay 549085':ab,ti OR 'bay43 9006':ab,ti OR 'bay43-9006':ab,ti OR 'bay439006':ab,ti OR 'bay54 9085':ab,ti OR 'bay549085':ab,ti OR 'fenesa':ab,ti OR 'hynap-sora':ab,ti OR 'nexavar':ab,ti OR 'reniloxa':ab,ti OR 'revamox':ab,ti OR 'rexanib':ab,ti OR 'sorafeb':ab,ti OR 'sorafenib tosilate':ab,ti OR 'sorafenib tosylate':ab,ti OR 'soratina':ab,ti OR 'weldinin':ab,ti OR 'sorafenib':ab,ti

#6:#4 OR #5

#7:'chemoembolization'/exp

#8:chemoembolization:ab,ti OR 'chemical embolization':ab,ti OR 'chemio-embolization':ab,ti OR 'chemo-embolisation':ab,ti OR 'chemo-embolization':ab,ti OR 'chemoembolisation':ab,ti OR 'chemoembolization, therapeutic':ab,ti OR 'therapeutic chemoembolization':ab,ti OR 'transcatheter chemoembolisation':ab,ti OR 'transcatheter chemoembolization':ab,ti OR 'transcatheter oily chemoembolisation':ab,ti OR 'transcatheter oily chemoembolization':ab,ti OR 'chemoembolization':ab,ti

#9:#7 OR #8

#10:'randomized controlled trial'/exp

#11:randomized controlled trial:ab,ti OR 'controlled trial, randomized’:ab,ti OR ‘randomised controlled study’:ab,ti OR ‘randomised controlled trial’:ab,ti OR ‘randomized controlled study’:ab,ti OR ‘trial, randomized controlled’:ab,ti OR ‘randomized controlled trial’:ab,ti

#12:#10 OR #11

#10:#3 AND #6 AND #9 AND #12

**Cochrane Library:69 records**

#1 MeSH descriptor: [Carcinoma, Hepatocellular] explode all trees

#2 (Carcinoma, Hepatocellular OR Carcinomas, Hepatocellular OR Hepatocellular Carcinomas OR Hepatocellular Carcinoma OR Hepatoma OR Hepatomas OR Liver Cancer, Adult OR Adult Liver Cancer OR Adult Liver Cancers OR Cancer, Adult Liver OR Cancers, Adult Liver OR Liver Cancers, Adult OR Liver Cell Carcinoma OR Carcinoma, Liver Cell OR Carcinomas, Liver Cell OR Cell Carcinoma, Liver OR Cell Carcinomas, Liver OR Liver Cell Carcinomas OR Liver Cell Carcinoma, Adult):ti,ab,kw

#3 #1 or #2

#4 MeSH descriptor: [Sorafenib] explode all trees

#5 (Sorafenib OR Sorafenib Tosylate OR Nexavar OR Sorafenib N-Oxide OR Sorafenib N Oxide):ti,ab,kw

#6 #4 or #5

#7 MeSH descriptor: [Chemoembolization, Therapeutic] explode all trees

#8 (Chemoembolization, Therapeutic OR Therapeutic Chemoembolization OR Chemoembolizations, Therapeutic OR Therapeutic Chemoembolizations):ti,ab,kw

#9 #7 or #8

#10 #3 and #6 and #9

**Web of Science: 61 records**

(TS=(Carcinoma, Hepatocellular) OR AB=(Carcinoma, Hepatocellular OR Carcinomas, Hepatocellular OR Hepatocellular Carcinomas OR Hepatocellular Carcinoma OR Hepatoma;Hepatomas OR Liver Cancer, Adult OR Adult Liver Cancer OR Adult Liver Cancers OR Cancer, Adult Liver OR Cancers, Adult Liver OR Liver Cancers, Adult OR Liver Cell Carcinoma OR Carcinoma, Liver Cell OR Carcinomas, Liver Cell OR Cell Carcinoma, Liver OR Cell Carcinomas, Liver OR Liver Cell Carcinomas OR Liver Cell Carcinoma, Adult)) AND (TS=(Sorafenib) OR AB=(Sorafenib OR BAY 43-9006 OR BAY 439006 OR BAY 43 9006 OR Sorafenib Tosylate OR 4-(4-(3-(4-Chloro-3-trifluoromethylphenyl)ureido)phenoxy)pyridine-2-carboxylic acid methyamide-4-methylbenzenesulfonate OR Nexavar OR BAY 545 9085 OR Sorafenib N-Oxide OR Sorafenib N Oxide)) AND (TS=(Chemoembolization, Therapeutic) OR AB=(Chemoembolization, Therapeutic OR Therapeutic Chemoembolization OR Chemoembolizations, Therapeutic OR Therapeutic Chemoembolizations)) AND (TS=(randomized controlled trial) OR AB=(randomized controlled trial OR controlled trial, randomized OR randomised controlled study OR randomised controlled trial OR randomized controlled study OR trial, randomized controlled OR randomized controlled trial))

**CNKI：323 records**

(Title+Keywords+Abstract: Hepatocellular carcinoma+Primary Liver Cancer+Liver Cancer+Liver Tumor) AND (Title+Keywords+Abstract: Sorafenib+Tosylate of Sorafenib) AND (Title+Keywords+Abstract: Hepatic Artery Chemoembolization + TACE）

**Wan-fang database：307 records**

Title or Keywords: (Hepatocellular Carcinoma or Primary Liver Cancer or Liver Cancer or Liver Tumor) and Title or Keywords: (Sorafenib or Tosylate Sorafenib) and Title or Keywords: (Hepatic Artery Chemoembolization or TACE)
