# Supplementary material for: Efficacy and safety of sorafenib combined with transarterial chemoembolization in the treatment of hepatocellular carcinoma: a meta-analysis of randomized controlled trials
Source: Front Oncol. 2025 Nov 10;15:1640879. doi: 10.3389/fonc.2025.1640879 (PMC12640826; doi:10.3389/fonc.2025.1640879)
Supplement: Supplementary file 2 [file DataSheet2.docx]

**Supplementary File S2** Intervention characteristics and outcome measures of the trials included in this meta-analysis.

| Studies | Liver cancer types | Intervention characteristics | PR(%) | ORR (%) | 12-month OS rates |
| --- | --- | --- | --- | --- | --- |
| Xinjian Wang *et.al* 2025 | primary HCC | TACE+Sorafenib  (400 mg twice daily) | 21(53.8) | 21(53.85) | 28 |
|  |  | TACE alone | 16(41.0) | 16(41.03) | 17 |
| Yunyun Jie *et.al* 2024 | intermediate-advanced HCC | Sorafenib(400 mg twice daily) started 3 d before TACE | 19(51.4) | 21(56.76) | NA |
|  |  | TACE alone | 12(32.4) | 12(32.43) | NA |
| Jiurong Zhu *et.al* 2024 | unresectable HCC | Sorafenib(400 mg twice daily) started 5 d after TACE | 10(41.7) | 13(54.17) | NA |
|  |  | TACE alone | 7(29.2) | 8(33.33) | NA |
| Wenzhe Fan *et.al* 2024 | recurrent intermediate-stage HCC | Sorafenib(400 mg twice daily) started 1 day before TACE | 35(43.2) | 65(80.2) | 73 |
|  |  | TACE alone | 30(37.0) | 47(58.0) | 58 |
| Daolin Zeng *et.al* 2024 | advanced HCC | TACE+Sorafenib  (400 mg twice daily) | 41(82.0) | 41(82) | NA |
|  |  | TACE alone | 39(78.0) | 39(78) | NA |
| Xiaocen Wei 2022 | HCC | Sorafenib(400 mg twice daily) started 3-5 d after TACE | 11(27.5) | 23(57.5) | 37 |
|  |  | TACE alone | 7(17.5) | 14(35.0) | 30 |
| Quanguo Liu *et.al* 2020 | advanced HCC | Sorafenib(400 mg twice daily) started 7 d after TACE | 29(49.2) | 33(55.9) | 41 |
|  |  | TACE alone | 21(35.6) | 22(37.3) | 30 |
| Haibo Zhu 2020 | primary HCC | Sorafenib(400 mg twice daily)  started 10 d before TACE | 12(52.2) | 22(95.6) | NA |
|  |  | TACE alone | 10(47.6) | 15(71.4) | NA |
| Jingjie Pan *et.al* 2019 | intermediate-advanced primary HCC | Sorafenib(400 mg twice daily)  started 1 d after TACE | 19(35.2) | 19(35.19) | NA |
|  |  | TACE alone | 15(28.3) | 15(28.30) | NA |
| Masatoshi Kudo *et.al* 2019 | unresectable HCC | Sorafenib(400 mg daily)  started 14-21d before TACE | 34(42.5) | 57(71.3) | 77 |
|  |  | TACE alone | 26(34.2) | 47(61.8) | 63 |
| Tim Meyer *et.al* 2017 | unresectable HCC | Sorafenib(400 mg twice daily)  started 1 d before TACE | 39(24.8) | 84(54) | NA |
|  |  | TACE alone | 45(28.8) | 81(52) | NA |
| Lei Li *et.al* 2017 | HCC | Sorafenib(400 mg twice daily)  started 3 d after TACE | 20(52.6) | 22(57.9) | NA |
|  |  | TACE alone | 10(27.0) | 10(27.0) | NA |
| Jiahang Xie *et.al* 2015 | progressive primary HCC | Sorafenib(400 mg twice daily)  started 7 d after TACE | 30(69.8) | 33(76.7) | 32 |
|  |  | TACE alone | 21(52.5) | 22(55.0) | 22 |
| Yong Tan *et.al* 2015 | primary large HCC | Sorafenib(400 mg twice daily)  started 3 d after TACE | 11(37.9) | 12(41.4) | NA |
|  |  | TACE alone | 4(14.3) | 4(14.3) | NA |
| Zhijian You *et.al* 2015 | intermediate-advanced HCC | Sorafenib(400 mg twice daily)  started 2-5 d after TACE | 13(15.9) | 13(15. 9) | 65 |
|  |  | TACE alone | 6(7.7) | 6(7.7) | 42 |
| Rengui Zhou *et.al* 2014 | advanced primary HCC | TACE+Sorafenib  (400 mg twice daily) | 25(52.1) | 25(52.1) | 43 |
|  |  | TACE alone | 16(33.3) | 16(33.3) | 28 |
| Heng Sun *et.al* 2014 | unresectable HCC | Sorafenib(400 mg twice daily)  started 3-7 d after TACE | 11(13.6) | 11(13.6) | 56 |
|  |  | TACE alone | 7(8.6) | 7(8.6) | 41 |
| Siming Chen *et.al* 2012 | primary HCC | Sorafenib(400 mg twice daily)  started 3-7 d after TACE | 15(53.6) | 16(57,1) | 25 |
|  |  | TACE alone | 7(25.0) | 7(25.0) | 18 |
| Haiying Jiang *et.al* 2010 | intermediate-advanced HCC | Sorafenib(400 mg twice daily)  started 5 d after TACE | 15(50.0) | 15(50) | 25 |
|  |  | TACE alone | 11(36.7) | 11(36.7) | 17 |

Note: Data are n (%) for categories; HCC: hepatocellular carcinoma; PR: partial response rate; ORR: objective response rate; 12-month OS rates: 12-month overall survival rates; TACE: Transarterial chemoembolization; NA: Not available.
