# Supplementary material for: Efficacy and safety of sorafenib combined with transarterial chemoembolization in the treatment of hepatocellular carcinoma: a meta-analysis of randomized controlled trials
Source: Front Oncol. 2025 Nov 10;15:1640879. doi: 10.3389/fonc.2025.1640879 (PMC12640826; doi:10.3389/fonc.2025.1640879)
Supplement: Supplementary file 3 [file DataSheet3.docx]

**Supplementary File S3** Methodological quality evaluation of eligible trials.

| Studies | Random sequence generation | Allocation concealment | Blinding of participants and personnel | Blinding of outcome assessment | Incomplete outcome data | Selective reporting | Other bias |
| --- | --- | --- | --- | --- | --- | --- | --- |
| Xinjian Wang *et.al* 2025 | Low risk | Low risk | Low risk | Unclear risk | Low risk | Low risk | Low risk |
| Yunyun Jie *et.al* 2024 | Low risk | Low risk | Low risk | Unclear risk | Low risk | Low risk | Low risk |
| Jiurong Zhu *et.al* 2024 | Unclear risk | Low risk | Low risk | Unclear risk | Low risk | Low risk | Low risk |
| Wenzhe Fan *et.al* 2024 | Low risk | High risk | Low risk | Unclear risk | Low risk | Low risk | Low risk |
| Daolin Zeng *et.al* 2024 | Low risk | Low risk | Low risk | Unclear risk | Low risk | Low risk | Low risk |
| Xiaocen Wei 2022 | Low risk | Low risk | Low risk | Unclear risk | Low risk | Low risk | Low risk |
| Quanguo Liu *et.al* 2020 | Low risk | Low risk | Low risk | Unclear risk | Low risk | Low risk | Low risk |
| Haibo Zhu 2020 | Unclear risk | Low risk | Low risk | Unclear risk | Low risk | Low risk | Low risk |
| Jingjie Pan *et.al* 2019 | Unclear risk | Low risk | Low risk | Unclear risk | Low risk | Low risk | Low risk |
| Masatoshi Kudo *et.al* 2019 | Low risk | High risk | Low risk | Unclear risk | Low risk | Low risk | Low risk |
| Tim Meyer *et.al* 2017 | Low risk | Low risk | Low risk | Low risk | Low risk | Low risk | Low risk |
| Lei Li *et.al* 2017 | Unclear risk | Low risk | Low risk | Unclear risk | Low risk | Low risk | Low risk |
| Jiahang Xie *et.al* 2015 | Low risk | Low risk | Low risk | Unclear risk | Low risk | Low risk | Low risk |
| Yong Tan *et.al* 2015 | Low risk | Low risk | Low risk | Unclear risk | Low risk | Low risk | Low risk |
| Zhijian You *et.al* 2015 | Low risk | Low risk | Low risk | Unclear risk | Low risk | Low risk | Low risk |
| Rengui Zhou *et.al* 2014 | Unclear risk | Low risk | Low risk | Unclear risk | Low risk | Low risk | Low risk |
| Heng Sun *et.al* 2014 | Low risk | Low risk | Low risk | Unclear risk | Low risk | Low risk | Low risk |
| Siming Chen *et.al* 2012 | Unclear risk | Low risk | Low risk | Unclear risk | Low risk | Low risk | Low risk |
| Haiying Jiang *et.al* 2010 | Low risk | Low risk | Low risk | Unclear risk | Low risk | Low risk | Low risk |
