# Supplementary material for: Efficacy and safety of sorafenib combined with transarterial chemoembolization in the treatment of hepatocellular carcinoma: a meta-analysis of randomized controlled trials
Source: Front Oncol. 2025 Nov 10;15:1640879. doi: 10.3389/fonc.2025.1640879 (PMC12640826; doi:10.3389/fonc.2025.1640879)
Supplement: Supplementary file 4 [file DataSheet4.docx]

**Supplementary File S4** Summary of adverse events occurring in either group of the trials included in this meta-analysis

| Studies | Group | Patients | AE | | | |
| --- | --- | --- | --- | --- | --- | --- |
|  |  |  | Hand and foot skin reaction | Abdominal pain or Diarrhea | Nausea or Vomit | Fever |
| Xinjian Wang *et.al* 2025 | Sorafenib + TACE | 39 | NA | NA | 24 | 20 |
|  | TACE alone | 39 | NA | NA | 20 | 23 |
| Yunyun Jie *et.al* 2024 | Sorafenib + TACE | 37 | 19 | 15 | NA | NA |
|  | TACE alone | 37 | 17 | 14 | NA | NA |
| Jiurong Zhu *et.al* 2024 | Sorafenib + TACE | 24 | 2 | 2 | 3 | 2 |
|  | TACE alone | 24 | 1 | 3 | 5 | 2 |
| Wenzhe Fan *et.al* 2024 | Sorafenib + TACE | 81 | 0 | 50 | 36 | 15 |
|  | TACE alone | 81 | 43 | 75 | 35 | 14 |
| Daolin Zeng *et.al* 2024 | Sorafenib + TACE | 50 | 3 | 0 | NA | 4 |
|  | TACE alone | 50 | 1 | 2 | NA | 2 |
| Xiaocen Wei 2022 | Sorafenib + TACE | 40 | NA | NA | NA | NA |
|  | TACE alone | 40 | NA | NA | NA | NA |
| Quanguo Liu *et.al* 2020 | Sorafenib + TACE | 59 | 2 | 10 | 33 | 24 |
|  | TACE alone | 59 | 19 | 26 | 39 | 20 |
| Haibo Zhu 2020 | Sorafenib + TACE | 23 | NA | 1 | 2 | 2 |
|  | TACE alone | 21 | NA | 2 | 2 | 0 |
| Jingjie Pan *et.al* 2019 | Sorafenib + TACE | 54 | NA | NA | 10 | NA |
|  | TACE alone | 53 | NA | NA | 9 | NA |
| Masatoshi Kudo *et.al* 2019 | Sorafenib + TACE | 80 | 0 | 0 | NA | 18 |
|  | TACE alone | 76 | 41 | 11 | NA | 15 |
| Tim Meyer *et.al* 2017 | Sorafenib + TACE | 157 | 0 | 16 | 2 | NA |
|  | TACE alone | 156 | 12 | 36 | 2 | NA |
| Lei Li *et.al* 2017 | Sorafenib + TACE | 38 | 16 | 14 | NA | NA |
|  | TACE alone | 37 | 19 | 18 | NA | NA |
| Jiahang Xie *et.al* 2015 | Sorafenib + TACE | 43 | 3 | 3 | 21 | 15 |
|  | TACE alone | 40 | 31 | 18 | 30 | 17 |
| Yong Tan *et.al* 2015 | Sorafenib + TACE | 29 | 0 | 0 | NA | NA |
|  | TACE alone | 28 | 2 | 1 | NA | NA |
| Zhijian You *et.al* 2015 | Sorafenib + TACE | 82 | 38 | NA | NA | NA |
|  | TACE alone | 78 | 41 | NA | NA | NA |
| Rengui Zhou *et.al* 2014 | Sorafenib + TACE | 48 | NA | NA | NA | NA |
|  | TACE alone | 48 | NA | NA | NA | NA |
| Heng Sun *et.al* 2014 | Sorafenib + TACE | 81 | 65 | NA | NA | NA |
|  | TACE alone | 81 | 63 | NA | NA | NA |
| Siming Chen *et.al* 2012 | Sorafenib + TACE | 28 | NA | NA | NA | NA |
|  | TACE alone | 28 | NA | NA | NA | NA |
| Haiying Jiang *et.al* 2010 | Sorafenib + TACE | 30 | 1 | 3 | NA | NA |
|  | TACE alone | 30 | 11 | 11 | NA | NA |

Note: AE: Adverse event; NA: Not available.
